# Influence of the Environment on Body Temperature of Racing Greyhounds

**DOI:** 10.3389/fvets.2016.00053

**Published:** 2016-06-30

**Authors:** Jane McNicholl, Gordon S. Howarth, Susan J. Hazel

**Affiliations:** ^1^School of Animal and Veterinary Sciences, University of Adelaide, Adelaide, SA, Australia

**Keywords:** greyhound, heat strain, heat stress, animal welfare, myoglobin, sport

## Abstract

Heat strain is a potential risk factor for racing greyhounds in hot climates. However, there have been limited studies into the incidence of heat strain (when excess heat causes physiological or pathological effects) in racing greyhounds. The aim of this study was to determine if heat strain occurs in racing greyhounds, and, if so, whether environmental factors (e.g., ambient temperature and relative humidity) or dog-related factors (e.g., sex, bodyweight, color) are associated with the risk of heat strain. A total of 229 greyhounds were included in over 46 race meetings and seven different race venues in South Australia, Australia. Rectal temperatures of dogs were measured pre- and postrace and urine samples collected for analysis of myoglobinuria. Ambient temperature at race times ranged between 11.0 and 40.8°C and relative humidity ranged from 17 to 92%. There was a mean increase in greyhound rectal temperature of 2.1°C (range 1.1–3.1°C). A small but significant association was present between ambient temperature and increase in rectal temperature (*r*^2^ = 0.033, *P* = 0.007). *The mean ambient temperature at race time, of dogs with postrace rectal temperature of or exceeding 41.5*°*C, was significantly greater than at race time of dogs with a postrace rectal temperature* ≤*41.5*°C *(31.2 vs. 27.3°C, respectively, P* = *0.004)*. When the ambient temperature reached 38^o^C, over one-third (39%) of dogs had a rectal temperature >41.5°C. Over half of postrace urine samples were positive by Dipstick reading for hemoglobin/myoglobin, and of 77 urine samples positive for Dipstick readings, 95% were positive for myoglobin. However, urinary myoglobin levels were not associated with ambient temperature or postrace rectal temperatures. The mean increase in rectal temperature was greater in dark (black, blue, brindle) than light (fawn and white) colored greyhounds. The results suggest heat strain occurs in racing greyhounds, evidenced by postrace rectal temperatures over 41.5°C and postrace myoglobinuria. Risk of heat strain may be increased in higher ambient temperatures and in darker colored greyhounds. Further research into the incidence of heat strain in racing greyhounds, and longer term physiological responses to heat strain, are warranted.

## Introduction

Regulation of body temperature is essential for maintenance of life. Vertebrates regulate body temperature by both behavioral and physiological means. In mammals, cutaneous thermal sensors measure surface temperature, while core temperature is measured in the spinal cord and areas of the brain (1, 2). Heat stress describes the environmental or metabolic factors impacting on the body when its thermoregulatory mechanisms are challenged, due either to excessive ambient temperatures or extreme heat production whereas heat strain is the resultant physiological or pathological effects (3). Heat stroke occurs when the body’s heat dissipating mechanisms are overwhelmed due to exposure to an environmental temperature exceeding body temperature (classic or environmental heat stroke) or when metabolic heat accumulates due to strenuous exercise (exertional heat stroke) (4). Heat stroke entails major organ failure and is life threatening (5, 6). In climates with high ambient temperatures, such as in Australia, heat stress and heat stroke are potential risk factors for dogs used in work and recreation, including the racing greyhound.

Dogs are able to maintain their temperature over a broad range of environmental and climatic conditions. Different thermoneutral zones have been estimated using a variety of methods and types of dog. A range of 23–27°C has been suggested as thermoneutral for three mixed breed dogs with bodyweights 8.5–10.5 kg (7), while for Innuit dogs it is −25 tο 10°C (8). In greyhounds, the thermoneutral zone has been estimated to be 16–24°C (9). Symptoms of heat illness in dogs include panting, dry mucous membranes, prolonged capillary refill time, ataxia, and elevated body temperature (10, 11).

As greyhounds have been subject to intense selection for athletic performance over several centuries (12, 13), and 60% of the body mass is muscle (14), the large locomotor muscles may contain high mitochondrial and capillary volumes as found in some athletic marsupials (15). It could therefore be expected greyhounds would generate heat at a high rate and be particularly susceptible to exertional hyperthermia. Muscular activity generates heat as a by-product of ATP production and utilization. When the ambient temperature nears or exceeds body temperature, heat can only be lost by evaporation, which in dogs is achieved *via* the respiratory tract (16). During strenuous exercise, the respiratory rate increases, thus facilitating heat transfer, however, high levels of humidity may restrict the amount of heat lost. Although the milder manifestations of heat illness described in humans, such as heat rash and heat edema, have not been described in dogs, more significant symptoms such as cramps and fatigue are commonly exhibited by greyhounds following even short periods of strenuous exercise (17, 18). The elevation in rectal and muscle temperature resulting from prolonged exercise by dogs is associated with reduced levels of high energy phosphates (ATP and CrP) and increased levels of muscle lactate, pyruvate, and AMP, which may contribute to fatigue (19). Although greyhounds racing in environmental temperatures of 42°C are thought to be at risk of heat stroke (20), there has been little research into the effects on greyhounds of running in high ambient temperatures.

Heat stroke has been recognized for centuries, but until relatively recently the mechanisms were poorly understood. Shapiro et al. (21) was able to demonstrate, using dogs (which do not sweat), that heat stroke was due to tissue damage resulting from elevated body temperature and not cessation of sweating as had been previously believed. A subsequent study in which anesthetized dogs were heated to a rectal temperature of 44.5°C, revealed increased levels of serum enzymes such as glutamic-pyruvic glutaminase (SGPT) and alkaline phosphatase in the terminal stages of hyperthermia, indicative of tissue damage (22). The authors also noted necrosis of liver and intestinal epithelium and turbid, brown urine, indicative of impaired renal function. Current understanding is that heat stroke involves impairment of cellular function, denaturing of proteins (both structural and enzymatic) and disruption of lipid membranes, similar to systemic inflammatory response syndrome (SIRS) (23). Hyperthermia induces intestinal ischemia and increased intestinal wall permeability, which permits leakage of endotoxins (10, 24).

Rhabdomyolysis (muscle breakdown) may be a consequence of both strenuous exercise (25, 26) and heat strain (27). Exercise-induced muscle fiber damage has been reported in humans (28) and animals (29–32). Following rhabdomyolysis, there is rapid release of cell breakdown products, such as the enzymes creatine kinase and lactate dehydrogenase; ions such as potassium and phosphate and muscle proteins such as myoglobin (25, 33). Muscle fiber damage may be detected by the presence of myoglobin in urine (myoglobinuria) (33). Myoglobin is nephrotoxic and in humans, elevated levels of myoglobin in serum or urine are associated with a risk of acute renal failure and subsequent mortality. Myoglobinuria has been reported in greyhounds (34) but the incidence is unknown and the levels of myoglobin excreted have not been quantified.

Dehydration has been identified by many authors as a precursor or precipitating factor for heat stroke in humans (35, 36). It has been widely accepted that dehydration of 2–3% is a major risk factor for heat illness and that a fluid deficit of as little as 1.5–2% may have a negative effect on performance (37). A decrease in plasma volume of 6 ± 2% increases the rate of heat accumulation in dogs exposed to high external heat load (38); furthermore, a significant elevation in rectal temperature occurs in dehydrated versus non-dehydrated dogs exercising on a treadmill at 25°C (39). Racing greyhounds may lose up to 6% of their bodyweight, due to dehydration, in the prerace kenneling period (40). Such losses might lead to increased risk of hyperthermia and increased risk of renal damage from myoglobinuria.

Phenotypical factors, such as sex and bodyweight, may also influence the response of greyhounds to environmental conditions. Sex-based differences in response to elevated temperatures and exercise occur in humans (41) and mice (42). Differences exist in the susceptibility of male and female rats to disruption of the sarcolemma following exercise (43), and there are apparent protective effects of estrogen against exercise-induced muscle damage in rats and humans (44, 45). Sex differences in susceptibility to exertional rhabdomyolysis in dogs have not been reported. Body weight is also important, as the basal metabolic rate of mammals and resultant heat production increases with bodyweight (46). With an increase in body mass there is a reduction in the ratio of body surface area to body mass and an increase in the distance from core to surface: both of these factors reduce the ability of an animal to dissipate heat and in exercising animals, may lead to greater heat accumulation (47). Finally, coat color is important as there is a widely held lay opinion that black greyhounds are more stressed by high ambient temperatures than other colored greyhounds, a belief which might be supported by studies in other species. The white winter coats of arctic species have greater reflectivity than darker summer coats (48) and in cattle, white coat color increases heat tolerance over brown or black (49).

Since this study is performed in Australia, it is important to provide some background on greyhound racing in this country. Greyhound racing is conducted in all Australian states and territories, each of which has a governing body, which administers and regulates racing. The population of racing greyhounds in South Australia is approximately 60% male and 40% female (T. Hayles, GRSA personal communication, June 2013). Sex limited races for greyhounds are seldom programed so the majority of races include animals of both sexes. Greyhounds commence racing at or above 16 months of age and generally have a racing career of 18–24 months, thus racing greyhounds have a relatively narrow age span of approximately 2–4 years of age. Five basic coat colors are recognized: black, blue (a dilute of black which can be pale to dark gray), brindle (dark stripes over a base color producing black brindle, blue brindle, red brindle, dun brindle, fawn brindle, dark brindle, light brindle), fawn (dark fawn, light fawn, red fawn, blue fawn, or dun fawn), and dun which may range from a light blue fawn, through a rich red fawn, to a deep rich chocolate color, with the dominating factor being a pink to brown colored nose leather. Dun is extremely rare. Any of these colors may be distributed over the body in patches over a white base, and such greyhounds are described as parti-colored.

No studies to date to our knowledge have investigated body temperature changes in greyhounds during racing in Australia. Furthermore, it is not known if potential risk factors, such as sex, body weight, or coat color, may alter the risk of heat strain. The aims of this study were to determine if:

1) body temperature increases more in greyhounds raced during hot and during humid days;
2) body temperature is influenced by dog sex, body weight, coat color, or cooling vest use;
3) myoglobin is present in urine in greyhounds following racing, and;
4) if myoglobin is present, if levels are affected by ambient temperature, race distance, dogs’ level of fitness, sex, body weight, or postexercise rectal temperature.

## Materials and Methods

An observational study was commenced in 2010 to record temperature and humidity at racing venues around South Australia and to record body temperature changes of greyhounds competing in races. The climate of the more populated districts of South Australia is described as Mediterranean with cool wet winters and hot dry summers (50). In summer, mean maximum daily temperature for the capital city, Adelaide (Latitude 34° 50′S–Longitude 138° 30′E) is 29°C (51). Animal ethics approval was provided for this study by the University of Adelaide Animal Ethics Committee.

### Ethic Statement

Owners or trainers gave informed signed consent at the racetrack to participate in the study.

### Venues

The pool of racing greyhounds in South Australia fluctuates, as dogs commence or terminate their racing careers and move between states. Across Australia in 2011, there were 43,259 races organized in 4068 race meetings and over $82,000,000 (AUS) prize money was distributed: the greyhound industry had 12,280 greyhounds registered for racing for a total of 330,429 starters (Greyhounds Australasia 2011). In South Australia, greyhound racing is controlled by Greyhound RacingSA (GRSA) and the industry represents over 16% of Totalizer Agency Board (TAB) market share and distribution, which in 2010–2011 was $10 m (AUS) (52).

Racing is conducted throughout the year. At the time of commencement of this study, there were eight racetrack venues, three of which (Barmera, Port Augusta, and Virginia) did not conduct race meetings during January and February. All racetracks with the exception of Virginia were oval tracks. The Virginia track was straight and only held lure coursing events. Race meetings were conducted three times per week at both Angle Park and Gawler but at approximately fortnightly intervals at other tracks.

On oval tracks, 7–12 races are programed per meeting and eight greyhounds (plus two reserves) are drawn to compete in each race. At lure coursing meetings, 6–10 events are held, each of which include 4–32 runners drawn to run off in pairs. Winners of each course proceed to the next round until the surviving pair contested a final. At the Virginia straight track, lure coursing meetings are only held between April and October and meetings commenced at 9.30 a.m. and finished by 2 p.m. At other tracks, races are scheduled between 12 p.m. and 11 p.m. and all greyhounds engaged at a meeting are presented for inspection prior to kenneling and kenneled at least 30 min prior to the first race on the program.

Pre-kenneling inspection entails an identity check of each greyhound by color, markings, ear tattoo, and/or microchip number. Every greyhound is then weighed and undergoes a brief veterinary inspection for health and racing suitability. Following inspection, each greyhound is confined in an allocated kennel until approximately 10 min prior to its race start time. At the Virginia racetrack, greyhounds are confined in their transport vehicles for the duration of the meeting.

Forty-six race meetings were attended at seven different venues and at different times of the year (Table 1). One track was straight (Virginia), and the other six were oval with various radii and circumferences. Two race tracks (Port Augusta and Virginia) have grass surfaces and five (Angle Park, Barmera, Gawler, Port Pirie, and Strathalbyn) have sand/loam surfaces. Races were conducted over distances of 300–731 m.

**Table 1:** **Racetrack venues attended during 2011**.

Track	January	February	March	April	May	June	July	August	September	October	November	December
Angle Park	1	3	2					1	1	1		4
Gawler	7	3	3		1			1	2		1	2
Strathalbyn	2	2										
Barmera			1	1				1	1	1		
Port Pirie		1										
Port Augusta			1									
Virginia				1	1							

### Environmental Monitoring

Ambient conditions inside the kennel houses and trackside were monitored with a weather station (La Crosse Technology, wireless 433-MHz Weather Station). Kennel house conditions were measured by the monitor placed approximately 1.2 m above ground level in the kennel area. Trackside conditions were measured by the outdoor monitor placed 1.2–1.8 m above ground level adjacent to the track, at a readily accessible location. Temperature and relative humidity were manually recorded at the start time of each race in which a selected greyhound was competing. Cloud cover was recorded in oktas on a scale 0–8 on which 0 = nil cloud and 8 = complete cloud cover (53). Some races were conducted after dusk, which was recorded as 10.

### Thermometers

Temperatures were measured using a rectal clinical veterinary thermometer (Vicks Speed Read digital thermometer).

### Cooling Jackets

The use of cooling jackets on greyhounds at racetracks, in temperatures >30°C, has been encouraged by GRSA for over 4 years (P. Marks, personal communication, January 2014). The jackets used during this study (Cool Champions, Silver Eagle Outfitters http://www.coolweave.com.au) were soaked in iced water for 30 min prior to initial use and also between uses.

### Animals

The population of racing greyhounds in South Australia is approximately 1200 animals (G. Barber, GRSA personal communication, January 2010). On the day prior to a race meeting, the fields were accessed online at http://sa.thedogs.com.au and greyhounds were selected by random draw using Excel RANDBETWEEN function. If two greyhounds in the care of one trainer at one meeting were selected, a draw was repeated to select an alternative greyhound. Details of the age, sex, color, sire, dam, and previous race history of each greyhound were recorded from the published race fields.

**Figure 1:**
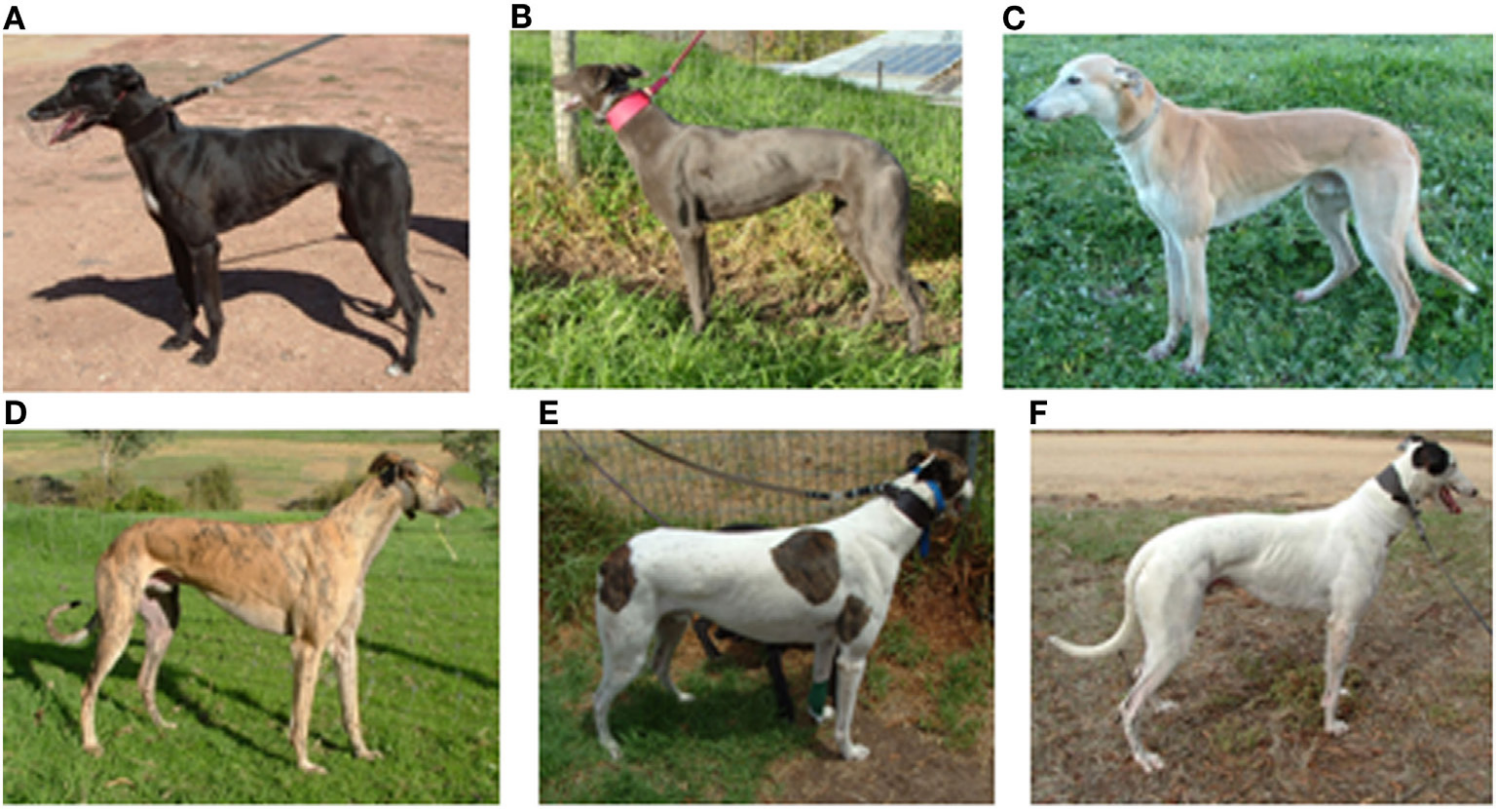
**Greyhound colors: (A) black, (B) blue, (C) fawn, (D) brindle, (E) parti-colored white and brindle, (F) parti-colored white and black**.

For the purpose of this study, greyhounds were assigned a fitness score expressed in meters from 300 to 700 m in 100-m increments. The fitness score was based on the mode of the greyhound’s last three races or trials to the nearest 100 m. From 246 races, 238 greyhounds were selected to participate in the study (134 males, 104 females) aged 18 months to 5 years (mean 2.6 years). In the greyhound industry, the generally accepted desirable weight range for racing dogs is 26–34 kg, for the purpose of analysis, the greyhounds were divided by bodyweight into four groups commonly used in the industry: <26, 26–30, >30–34, >34 kg. Any dog in with more than 50% white coat color was classified as white, thus creating five color groups (Figure 1). One greyhound was selected three times, and six greyhounds were selected twice. For the purpose of analysis, each of these greyhound’s race starts was treated as a separate data point.

### Procedure

At each race meeting, approximately 30 min prior to the commencement of greyhound admission, a list of selected greyhounds was provided to the stewards and a copy was posted in a prominent position at the kennel house entrance. As the selected greyhounds were presented for identification checks, the trainer was advised of the selection and permission sought for inclusion in the study. Some trainers declined to include some of the pre-selected greyhounds because of perceived temperamental unsuitability. Each participating greyhound was weighed and then had its temperature recorded as arrival temperature. Greyhounds were subsequently confined in their allocated kennels (or at lure coursing meetings, in trailers) up until approximately 10 min prior to their race start time.

Each greyhound was then collected from its allocated kennel and had a racing vest fitted, underwent identity check by stewards and was then taken outdoors to relieve itself. Urine samples were collected by voluntary voiding from 182 greyhounds. Rectal temperature was then recorded (prerace temperature). After completion of a race, greyhounds were collected by their handlers and returned to the kennel house and each greyhound’s temperature was again recorded (postrace temperature). This time point was between 2 and 3 min after greyhounds ceased to run. All greyhounds then underwent hosing and were allowed to drink from a hose prior to being returned to their allocated kennels. The greyhounds, which competed in lure coursing events, were assessed after two courses, which were conducted at least 30 min apart, during which time the greyhounds were lightly hosed with cold water, offered cold water to drink, and confined in well ventilated trailers, thus permitting cooling. An attempt was made to follow up each greyhound to collect a second urine sample before the greyhound left the racetrack. Postrace urine samples were collected from 203 greyhounds. Rectal temperature before and after racing was obtained for 229 dogs (131 males, 98 females).

### Urinalysis

Matched pre- and postrace urine samples were collected by voluntary voiding from 177 greyhounds, 104 males, 73 females: (a) in the immediate prerace exercise period, and (b) as the greyhounds were leaving the track. Postrace samples were obtained between 30 min and 3 h after racing, dependent on the trainer’s schedule. Urine samples were transferred from the collecting ladle into specimen containers immediately after collection and placed on ice for transport, then refrigerated up to 12 h prior to screening. Urine samples were centrifuged at 3000 rpm for 5 min to remove red blood cells. Reagent strips (Siemens Multistix 10 SG) were then immersed in the supernatant urine and read following the manufacturer’s protocol. Results for blood/hemoglobin are expressed as trace, 1+, 2+, 3+ (1+ equivalent to 0.030–0.065 mg/dl). The manufacturer states the test is equally sensitive to myoglobin and hemoglobin. Samples were then stored frozen at −20°C for up to 12 months. Subsequently, 87 urine samples (77 which tested positive for blood/hemoglobin, 7 which tested negative and 3 unknown) were thawed and subjected to enzyme linked immunosorbent assay (ELISA) using Dog Myoglobin (Life Diagnostics, Inc.) and read at 450 nm (Benchmark Plus BIO-RAD).

### Data Analysis

Data for the analysis of rectal temperature changes and associations with ambient temperature and humidity was analyzed with GraphPad Prism 6. Linear regression analysis was conducted to determine the association between shade temperature and relative humidity on rectal temperature at three time points: (a) on arrival; (b) pre-race; and (c) postrace. Linear regression analysis was also used to determine any association between ambient temperature, race distance, dogs’ level of fitness, bodyweight, and postexercise body temperature and postexercise urine levels of myoglobin. Data were inspected for normality in distribution using the D’Agostino–Pearson test. Unpaired *t*-tests with Welches correction were conducted to determine sex-based differences in urine myoglobin, and the effects of cooling jackets worn postrace.

Statistical analyses to analyze effects of dog sex, weight, and color on rectal temperature changes were conducted using SPSS version 21. Data were inspected for normality in distribution using the D’Agostino–Pearson test. A mixed model which included the fixed effects of sex and color and covariate of weight and sire as a random term was fitted to the increase in rectal temperature and the postrace rectal temperature data. There was no sire variance thus a general linear model was fitted to the data with the above fixed effects and covariate. Any significant two-way interactions were retained in the model. A level of significance of *P* < 0.05 was used throughout.

## Results

### Environmental Conditions

**Figure 2:**
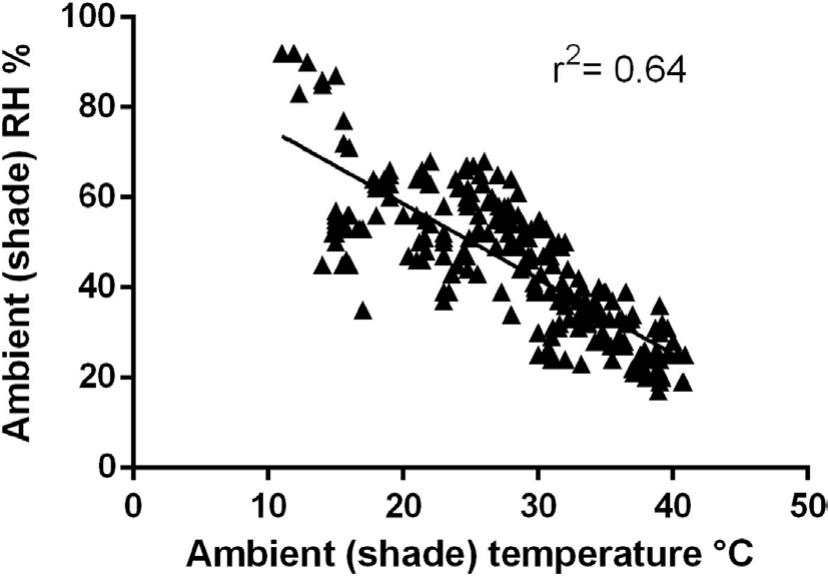
**Relationship between ambient temperature and relative humidity recorded at racetracks (*r*^2^ = 0.64, *p* = 0.0001)**.

Ambient (shade) temperature at each dog race start ranged from 11.0 to 40.8°C. Relative humidity ranged between 17 and 92%. As ambient temperature increased, relative humidity decreased (Figure 2, *r*^2^ = 0.64, *P* = 0.0001).

### Body Temperature

**Figure 3:**
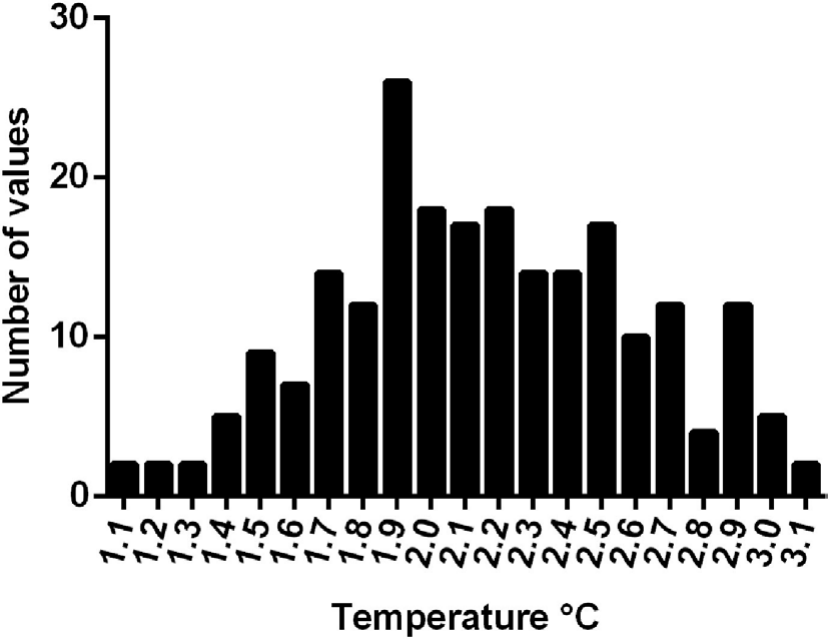
**Increase in rectal temperature from pre- to postrace in 229 greyhounds**.

Mean rectal temperature of 229 greyhounds on arrival at the race meetings was 39.2°C ± 0.5°C (range 38.2–40.5°C). Postrace, there was an increase in rectal temperature in all dogs. Mean postrace temperature was 41.0± 0.5°C (range 39.7–42.1°C) with a mean increase of 2.1°C (SD 0.4°C, range 1.1–3.1°C; Figure 3).

**Figure 4:**
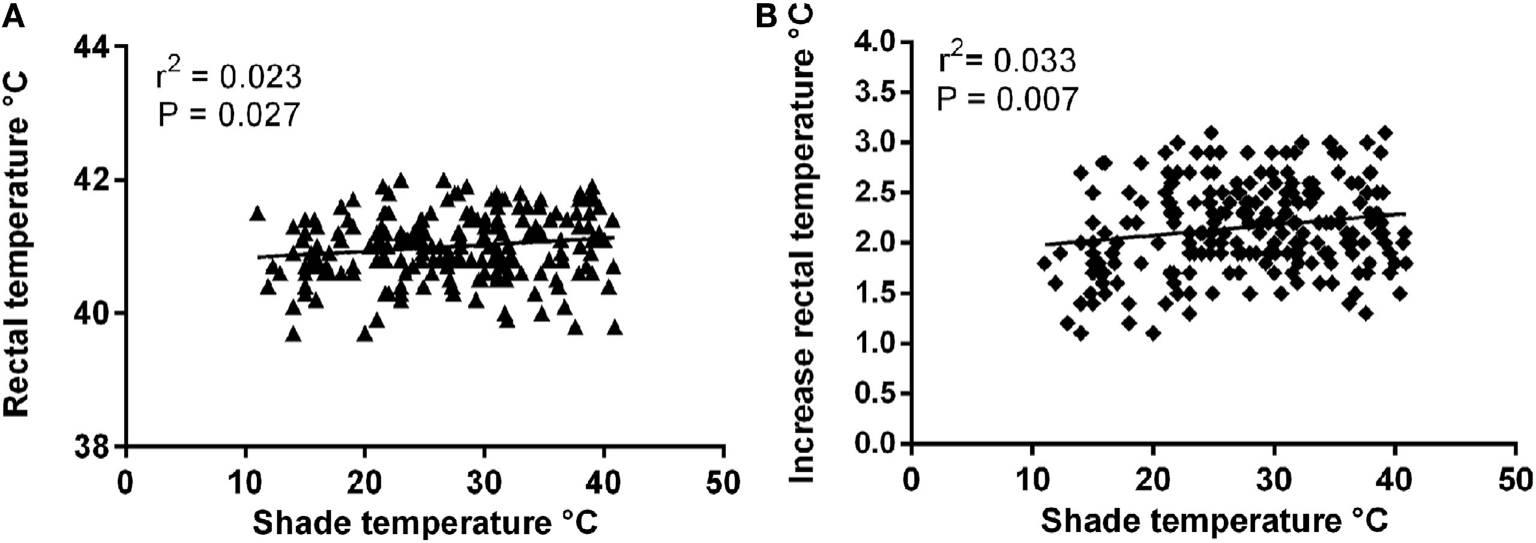
**Relationship between ambient temperature and: (A) postrace rectal temperature and (B) increase in rectal temperature**.

There was no significant effect of shade (ambient) temperature on rectal temperature on arrival (*r*^2^ = 4.5 × 10^−7^, *P* = 0.99). Postrace there was a small but significant relationship between shade temperature and both rectal temperature (*r*^2^ = 0.023, *P* = 0.03) and increase in rectal temperature (*r*^2^ = 0.033, *P* = 0.007; Figure 4). A significant inverse relationship between prerace rectal temperature and the increase in rectal temperature after racing was determined (*r*^2^ = −0.15, *P* = 0.001).

#### Effect of Relative Humidity

There was no significant association between relative humidity and postrace rectal temperature (*r*^2^ = 0.02, *P* = 0.06).

#### Effect of Race Distance

No association between race distances and levels of postrace rectal temperature was found (*r*^2^ = 0.004, *P* = 0.4).

#### Effect of Dog Fitness

No association was detected between dogs’ levels of fitness and levels of postrace rectal temperatures (*r*^2^ = 0.001, *P* = 0.7).

### Cooling Jackets

Dogs that wore cooling jackets (*N* = 41) had a significantly higher rectal temperature postrace compared to those that did not (*N* = 80; mean 41.19 ± SEM 0.06°C versus 41.01 ± SEM 0.06°C, respectively, *P* = 0.04; unpaired *t*-test with Welch’s correction).

### Critical Temperatures

As 41.5°C has been suggested as a critical body temperature for precipitating heat illness in dogs (11, 54), animals were allocated into two groups using a postrace rectal temperature delimiter of >41.5°C. The mean ambient temperature at race time of dogs with postrace rectal temperature >41.5°C was significantly greater (31.2°C ± SEM 1.0°C, *N* = 40) than at race time of dogs recording a rectal temperature ≤41.5 (27.3°C ± SEM 0.5°C, *N* = 189, unpaired *t*-test, *t* = 2.9, df = 227, *P* = 0.004).

**Figure 5:**
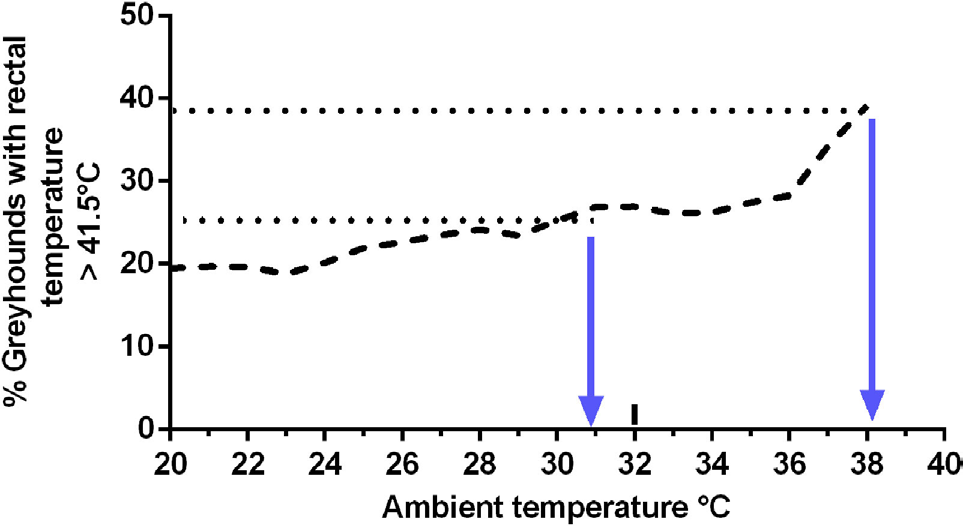
**Relationship between ambient temperature and the percentage of greyhounds with postrace rectal temperature >41.5°C**.

The percentage of greyhounds with postrace rectal temperatures of >41.5°C was plotted against ambient temperature (Figure 5). When the ambient temperature was 38°C, 39% of dogs had a rectal temperature of >41.5°C.

### Urinalysis

In both males and females, over half (100/177, 57%) of postrace urine samples provided positive dipstick readings for hemoglobin/myoglobin (Tables 2 and 3). There were more positive dipstick results for males versus females.

**Table 2:** **Results of dipstick test for blood, hemoglobin, or myoglobin in postrace urine samples**.

	Screened	Total positive hemoglobin	≥2^6^ ng/ml hemoglobin
Male	104	70 (67%)	47 (45%)
Female	73	30 (41%)	24 (33%)

**Table 3:** **Myoglobin results from 87 greyhound urine samples**.

Dipstick test result		Positive myoglobin^a^	Negative myoglobin
Positive blood/hemoglobin	77	73 (95%)	4 (5%)
Negative blood/hemoglobin	7	3 (43%)	4 (57%)
Unknown blood/hemoglobin	3	2 (67%)	1 (33%)

*^a^The lowest positive value was 5.8 ng/ml*.

Myoglobin levels detected in postrace urine samples ranged from 3 to 402 ng/ml (mean 93.3 ± 8.6 ng/ml). A significant association between dog bodyweight and myoglobin levels was detected (*r*^2^ = 0.05, *P* = 0.05). As variances of male and female myoglobin levels differed (*P* = 0.0003) an unpaired *t*-test with Welches correction was conducted. A significant difference in myoglobin levels between males and females was detected (male 73.17 ± SEM 7.76 ng/ml, female 128.20 ± SEM 20.15 ng/ml, *P* = 0.01). Linear regression analysis showed no significant associations between urinary myoglobin levels and ambient temperature, race distance, level of fitness, or postrace rectal temperature.

### Effect of Greyhound Color

The most common coat color of the included greyhounds was black (*N* = 115), followed by white (*N* = 37), with similar numbers of blue (*N* = 23), Brindle (*N* = 28), and Fawn (*N* = 26) colors. There was no significant difference in arrival or prerace rectal temperature of the five color groups (*P* = 0.5). However, mean postrace temperatures of the black, blue, and brindle greyhounds were 41.1 ± 0.4°C, 41.1 ± 0.5°C, and 41.1 ± 0.4°C, respectively, which were significantly higher than the fawn (40.9 ± 0.5°C) and white (40.8 ± 0.5°C; all *P* < 0.05) greyhounds. When the dogs were grouped into dark (black, blue, brindle) and light (fawn and white), the mean increase in temperature of the dark-colored dogs (2.2 ± 0.4°C) was significantly greater than the mean increase in temperature of the light-colored dogs (2.0 ± 0.4°C, *P* = 0.005). Postrace rectal temperatures of greyhounds racing in fully overcast/dark conditions (*N* = 31), or sunlight (*N* = 131) showed no significant difference (*P* = 0.5).

### Effect of Sex

There was no significant sex-related difference in arrival rectal temperature for 128 males and 101 females (*P* = 0.897). Prerace temperatures for males (*N* = 132) and females (*N* = 100) also did not differ (38.9 ± 0.5°C versus 38.8°± 0.3°C respectively, *P* = 0.46). A significant difference was found in postrace rectal temperature of male (*N* = 131) and female (*N* = 98) greyhounds (*P* = 0.004). Mean male postrace temperature was 41.1 ± 0.5°C, and mean female postrace temperature was 40.9 ± 0.4°C.

### Effect of Bodyweight

In the greyhound industry, the generally accepted desirable weight range for racing dogs is 26–34 kg: 73% of the selected greyhounds were within this range. The mean bodyweight was 30.2 ± 3.4 kg. Mean male bodyweight was 32.5 kg (median 32.0 kg), and mean female bodyweight was 27.2 kg (median 27.0 kg). There were no males in the <26 kg weight group, and no females in the >34 kg weight group (Table 4).

**Table 4:** **Sex distribution in four body weight groups of selected greyhounds**.

	<26 kg		26–30 kg		>30–34 kg		>34 kg	
	Male	Female	Male	Female	Male	Female	Male	Female
Number of dogs	0	23	10	68	82	7	39	0

**Figure 6:**
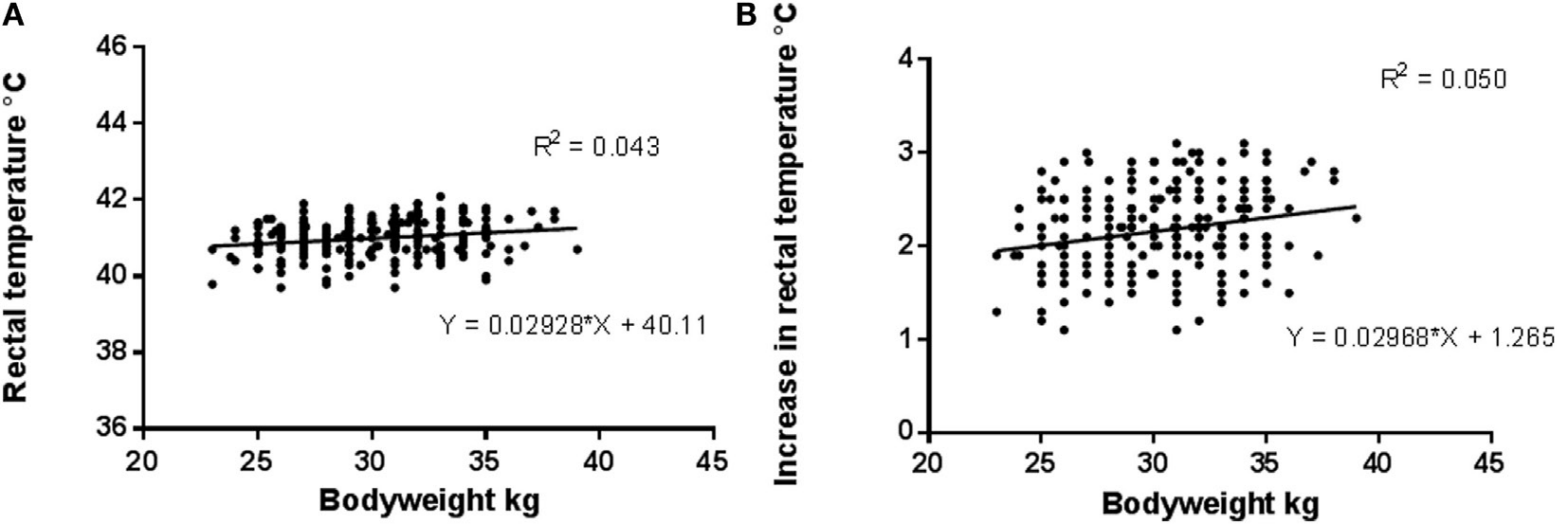
**Relationship between bodyweight and (A) postrace rectal temperature (*r*^2^ = 0.043, *P* = 0.009) and (B) increase in rectal temperature (*r*^2^ = 0.05, *P* = 0.006) after racing**.

A significant effect of bodyweight was noted on both actual rectal temperature (*r*^2^ = 0.043, *P* = 0.009) and the increase in rectal temperature (*r*^2^ = 0.05, *P* = 0.006) following racing (Figure 6).

## Discussion

The aims of the current study were to determine the body temperature responses to racing in greyhounds in South Australia. The study was designed to determine changes in rectal temperature following racing, and if any associations were present between increase in rectal temperature and environment factors, such as ambient temperature and relative humidity, and dog-related factors, such as sex, bodyweight, and color. Urinary myoglobin was measured postrace to determine if any pathological changes occurred and were related to heat strain.

In the current study, greyhounds competed in temperatures between 11 and 40°C. The mean increase in rectal temperature of 2.1°C was remarkable in view of the short duration of the periods of exercise. Although, as greyhounds expend almost as much energy in the first 7.5 s of a race as in the subsequent 22 s (55), it is not surprising that body temperature increases markedly in a short period of time. Although differences of up to 2°C between muscle and rectal temperatures have been measured in horses after 50-min exercise (56), the difference was less than 1°C after 10 min exercise as completed by the greyhounds in this study. Indeed in dogs, postexercise rectal temperatures higher than core temperature may result from the heat generated by the major muscles of the hind quarters (57). Most of the studies on hyperthermia in human and equine athletes have focused on hyperthermia as a result of prolonged periods of exercise. However, Drobatz and Macintyre (58) in their review of 42 clinical cases of heatstroke in dogs, remarked on the degree of morbidity after relatively short (20–30 min) periods of exercise, suggesting a high degree of susceptibility for dogs, compared to other species. As heat storage has been shown to be the principle limiting factor to intense exercise in cheetahs (59), it is probable that heat storage might similarly be a limiting factor to sprinting performance in greyhounds.

In the current study, the period of strenuous exercise was between 15 and 45 s for distances from 300 to 730 m. Additional activity was low intensity and was restricted to a total period of <15 min during which greyhounds were removed from holding kennels, approximately 10 min prior to scheduled race start time and walked to the starting boxes, 2 min prior to the race. Greyhounds exhibited varying levels of excitement in the prerace period, demonstrated by fine tremors or vigorous activity such as pulling or bouncing. The muscular activity involved in such behavior would generate heat and may have contributed to the increases in rectal temperature recorded. Greyhounds, bred and trained for racing, may develop an increase in rectal temperature (from resting levels) due to anticipation of activity (60). The negative association between prerace rectal temperature and increase in rectal temperature found in the current study illustrates the effectiveness of the thermoregulatory system, even under significant challenge.

### Environmental Conditions and Body Temperature

The current study revealed a small but positive association between ambient temperature and postexercise body temperature. These findings are in accord with those of Bjotvedt et al. (20) where greyhounds performing in temperatures above 107°F (42.0°C) were at risk of heat stroke. Although intense exercise is generally estimated to cause an increase in metabolic rate of 10–14 times the basal metabolic rate (61), increases in metabolic rate of up to 25 times BMR have been recorded in some canine species (8, 62). Dissipation of the heat generated may pose a particular challenge. Susceptibility to heat illness may vary between breeds of dog as ambient temperature has not been shown to affect rectal temperature in exercising Labrador retrievers (63), although the Labradors were only exercised in a temperature range of 11–28°C. In contrast, a significant association between ambient temperature and rectal temperatures is present in sled dogs working in ambient temperatures between −9 and 25°C (64).

No significant effect of relative humidity on rectal temperature was demonstrated in the current study. However, as the climate of South Australia is described as Mediterranean (50), with an inverse relationship between temperature and humidity, days with concurrent elevation of both factors are rare. In areas with a tropical climate, humidity might impose greater challenges. In exercising horses, the rate of increase in temperature of blood, measured in the pulmonary artery, is significantly higher in hot, humid conditions than in either hot or cold, dry conditions (65).

It may be concluded that racing, or undertaking equivalent intense exercise, in hot weather carries an increased risk of greyhounds developing heat illness. The risk increases notably in ambient temperatures ≥38°C. However, under current management systems in South Australia, no racing greyhounds were found to suffer from heat stroke. The greyhound racing industry in Australia has a number of “Heat” or “Hot Weather” policies, which vary between states and lack a consistent threshold temperature. It would be prudent to set the threshold for “Hot Weather Policies” at 38°C, at which temperature, changes to race programing should be made and stringent management procedures be implemented for greyhounds participating in races or trials.

### Critical Temperature

Many authors consider a rectal temperature ≥41.5°C to be a critical level for initiation of heat illness in dogs (11, 54, 58, 66, 67). During the current study, 45 greyhounds recorded a postrace rectal temperature >41.5°C which if not reduced, would place them at risk of heat illness. The mean ambient temperature at the time of these races was 31.2°C, which was 4°C greater than the mean ambient temperature at race time for greyhounds recording a rectal temperature <41.5°C. Therefore, 31°C might represent a threshold for risk estimation for heat stress in racing greyhounds. Such a threshold might be broadly accepted by participants in the greyhound racing industry, as there is a common perception amongst trainers of greyhounds that, at ambient temperatures >30°C, the animals show signs of thermal stress such as panting, which concurs with evidence from experimental settings (68). However, as in South Australia there are more than 80 days in summer with maximum daily temperature >30°C (51) setting 31°C as a threshold for canceling race meetings would represent major disruption to the industry. It has been suggested 36°C is the temperature at which thermal equilibrium can only just be maintained by dogs (69). In the current study, the percentage of greyhounds recording postrace rectal temperatures of ≥41.5° increased in gradual linear fashion up to 36°C and with a sharp rise when ambient temperatures reached 38°C. As 38°C is within the normal range of body temperature for dogs, the sharp increase in the number of greyhounds with temperature >41.5°C when ambient temperatures neared 38°C is in accord with the widely accepted view that, in environments at or above body temperature, thermoregulation is difficult.

### Cooling Jackets

The use of cooling jackets on greyhounds at racetracks has been fairly limited. During the current study, there appeared to be reluctance by some trainers to use them, either because of a belief that they were uncomfortable for the dogs or because of a perception the time taken to don the jackets was wasted. Results of this study revealed the unexpected finding that the mean rectal temperature of dogs wearing jackets postrace was slightly higher than those dogs which did not. Cooling jackets of a different type have been demonstrated to be effective in reducing the duration of postexercise hyperthermia in military dogs (70). Pre-competition use of ice jackets has been effective in reducing the degree of body heating in human athletes (71), but postexercise use of ice jackets is not advantageous in hyperthermic athletes (72). Further research into their use in greyhounds during hot conditions is warranted.

Alternative methods of estimating thermal stress in racing greyhounds might include panting score such as used for sheep (73) and cattle (49). However, in dogs panting is utilized not only to maintain homeothermy (74) but also as a result of exercise (19, 75), arousal (60), or anxiety (76). As this study was conducted at racetracks, all of the above factors could have influenced panting rate, and it was not practical to utilize panting rate as an indicator of heat stress.

### Urinalysis

Rhabdomyolysis has been recorded as a result of strenuous exercise in greyhounds (77, 78) and rhabdomyolysis may also result from hyperthermia (79). It could therefore be expected that greyhounds undertaking strenuous exercise in hot conditions would be at increased risk of developing rhabdomyolysis and myoglobinuria. Myoglobin is a small heme protein, which is released into plasma after muscle fiber rupture; plasma levels fall rapidly, as it is excreted into urine (79). As myoglobin is recognized as being nephrotoxic (33), it is possible that repeated exposure to significant levels would have a cumulative effect and that such exposure might contribute to the high incidence of renal disease seen in greyhounds (D. Fegan, personal communication, 2013).

### Effect of Phenotypic Factors

This study showed that postexercise temperature was influenced by several phenotypic factors. A significant though weak relationship was found between bodyweight and postexercise rectal temperature and also between bodyweight and the increase in rectal temperature. Coat color was also found to have a significant association with postexercise temperature, with greyhounds of dark colors developing higher rectal temperatures than light colored greyhounds. Many greyhound trainers believe that black greyhounds are more susceptible to heat stress than other colored dogs, as the trainers can feel temperature differences on the surface of their greyhounds. The finding that dark colored (black, blue, brindle) greyhounds develop higher temperatures than light colored (fawn and predominantly white) greyhounds is in keeping with findings in other species. Three naturally occurring color morphs of antelope have differences in core temperature (80) and McManus et al. (82) who examined the tolerance of different breeds and colors of sheep, to heat stress in Brazil, concluded that breed, coat type (wool/hair) and coat color were important modifiers. A number of studies of production animals have revealed that, under heat stress, white coated animals can maintain lower body temperatures than dark colored conspecifics and that coat color influences heat tolerance (81, 82). This has been attributed to the greater reflectivity of the white coats, leading to less heat accumulation. In the current study, it was anticipated that dark coated greyhounds, racing in sunlight, might develop higher body temperatures than those racing in shaded or dark conditions, however no significant effect of sunlight was detected. It seems therefore, that direct solar radiation was not a significant contributor to the temperatures recorded. However, thermal radiation from the track surface and surroundings may have contributed to the higher temperatures recorded in dark coated dogs.

### Sex

No significant differences between sexes were recorded for rectal temperatures either on arrival or prerace. However, males had higher postrace and mean increases in rectal temperature than females. Sex based differences in body temperature could be a result of sex hormones, body proportions, or thermoregulatory mechanisms. Gender differences in response to thermal and exercise challenges have been reported in humans (41, 83). The principle mechanism of heat loss in humans is sweating and considerable efforts have been directed at examining differences in sweating and sudomotor responses (84, 85). However, as sweating is not utilized as a heat loss mechanism in canines, it is not valid to attempt to extrapolate from such studies. The influence of estrogen and progesterone on core temperature during the menstrual cycle of women has long been recognized (86). Complex interactions between norepinephrine and estrogen occur in the brain of women, whereby estrogen raises the sweating threshold and norepinephrine narrows the thermoneutral zone by initiating heat dissipation (87). Hanada et al. (42) identified receptor activator of necrosis factor kB ligand (RANKL) and its tumor necrosis factor receptor (RANK) as key factors of central control of thermoregulation in female but not male mice and suggested that, in murine species, female thermoregulation is, in part, regulated by ovarian sex hormones (42).

The higher levels of myoglobinuria found in female greyhounds was unexpected, as it has been reported that female animals suffer less muscle damage than males (88). However, female horses are reported to be more frequently affected by exertional rhabdomyolysis than males (89, 90). Female rats are less susceptible to exercise-induced muscle damage than males (43, 91), likely due to a protective effect of estrogen (92). However, similar protection is unlikely to have occurred in greyhounds during the current study as testosterone proprionate was permitted to prevent estrous in female greyhounds at that time (93). In Australia, female greyhounds are not permitted to race whilst in estrous or for 28 days post estrous (93) and reduced performance in the diestrous period of up to 91 days has been reported (94). Therefore, racing female greyhounds can be assumed to be in late diestrous or anestrous with relatively low levels of circulating estrogen and progesterone (95). Alternatively, there may be a species related difference in response to exercise or even a breed specific response, as greyhounds exhibit other physiological and hematological variations from other breeds of dog (96–98). During the current study, no data were collected on the use of testosterone or other permitted hormones nor on the natural hormonal status of the females, so further studies on the responses to exercise of male and female greyhounds are required.

### Bodyweight

Many of the studies on thermoregulation and exercise in humans have investigated the differences, which might be attributable to anthropometric features such as body proportions and fat distribution (69, 99, 100). Lean animals may dissipate heat more readily than obese animals, as in the latter, sub-cutaneous fat impedes heat transfer to the environment (101). However, during exercise, both muscle and core temperature increase more in lean than obese rats (102). Almost all of the greyhounds in this study had a body condition score of 2 and variations in body fat are unlikely to have affected the results.

The positive associations between bodyweight and both postrace and increase in rectal temperature may be attributed to the amount of energy utilized during activity. As the energy requirements to move a body increase with an increase in bodyweight (103), metabolic heat production also rises. In both birds and mammals, the energetic cost of exercise is related to both body mass and speed (104, 105). In humans, metabolic heat production resultant from muscle contraction creates an internal heat load proportional to exercise intensity (106, 107). Although the periods of exercise of the greyhounds were limited to <45 s at maximum effort, as greyhounds have a high proportion of muscle (14) and the rate of heat accumulation in muscle increases with intensity of work (47), it is apparent that greyhounds exercising at maximum effort generate a very high heat load. In a recent study, rats selected for high capacity running, exhibited high levels of energy expenditure and muscle heat dissipation (108). The authors suggested that these effects might be due to intrinsic aerobic capacity and that similar expression of skeletal muscle proteins might be found in other species. Greyhound muscle exhibits a high rate of anaerobic glycogenolysis (109); further research is warranted in the area of greyhound muscle energetics and heat production.

## Conclusion

It may be concluded that racing, or undertaking equivalent intense exercise, in hot weather carries an increased risk of greyhounds developing heat illness. The risk increases notably in ambient temperatures ≥38°C. Large, dark-colored greyhounds are at greater risk of developing high body temperature than small, light-colored greyhounds, when undertaking strenuous exercise in hot conditions. Pre- and postexercise cooling should therefore be applied with particular care to large black, blue, or brindle greyhounds to prevent development of heat strain. The greyhound racing industry in Australia has a number of “Heat” or “Hot Weather” policies which vary between states and lack a consistent threshold temperature. It would therefore be prudent to set the threshold for “Hot Weather Policies” at 38°C, at which temperature, changes to race programing should be made and stringent management procedures be implemented for greyhounds participating in races or trials. Further research is required to investigate environmental effects on greyhounds racing in tropical climates.

## Author Contributions

JM was responsible for conception and design of the work, the acquisition, analysis and interpretation of data, drafting and revising, and gave final approval for it to be published. GH was responsible for help in interpretation of data and revising, and gave final approval for it to be published. SH assisted in the conception and design of the work, the acquisition, analysis and interpretation of data, drafting and revising, and gave final approval for it to be published. All authors agree to be accountable for all aspects of the work in ensuring that questions related to the accuracy or integrity of any part of the work are appropriately investigated and resolved.

## Conflict of Interest Statement

Funding for this study was provided by Greyhound Racing South Australia and JM is employed as a racetrack veterinarian for greyhound races in South Australia. The authors believe that neither of these perceived conflicts influenced the design, analysis and interpretation of the data presented.
